# Current Evidence for Immune Checkpoint Inhibition in Advanced Hepatocellular Carcinoma

**DOI:** 10.3390/curroncol30090628

**Published:** 2023-09-21

**Authors:** Victoria Foy, Mairéad G. McNamara, Juan W. Valle, Angela Lamarca, Julien Edeline, Richard A. Hubner

**Affiliations:** 1 Department of Medical Oncology, The Christie NHS Foundation Trust, Wilmslow Rd., Manchester M20 4BX, UK; 2 Division of Cancer Sciences, University of Manchester, Oxford Rd., Manchester M13 9PL, UK; 3 Department of Oncology, OncoHealth Institute, Fundación Jiménez Díaz University Hospital, Avenida de los Reyes Catolicos 2, 28040 Madrid, Spain; 4 Centre Eugène Marquis, Av. de la Bataille Flandres Dunkerque-CS 44229, CEDEX, 35042 Rennes, France; j.edeline@rennes.unicancer.fr

**Keywords:** ICI, HCC, PD-L1, PD-1, immunotherapy, biomarkers

## Abstract

The treatment of advanced unresectable HCC (aHCC) remains a clinical challenge, with limited therapeutic options and poor prognosis. The results of IMbrave150 and HIMALAYA have changed the treatment paradigm for HCC and established immune checkpoint inhibition (ICI), either combined with anti-angiogenic therapy or dual ICI, as preferred first-line therapy for eligible patients with aHCC. Numerous other combination regimens involving ICI are under investigation with the aim of improving the tumour response and survival of patients with all stages of HCC. This review will explore the current evidence for ICI in patients with advanced HCC and discuss future directions, including the unmet clinical need for predictive biomarkers to facilitate patient selection, the effects of cirrhosis aetiology on response to ICI, and the safety of its use in patients with impaired liver function.

## 1. Introduction

Primary liver cancer is the sixth most commonly diagnosed cancer globally and the third leading cause of cancer-related death [1]. Hepatocellular carcinoma (HCC) compromises 75–85% of primary liver cancers and is associated with chronic liver inflammation and subsequent fibrosis and cirrhosis [1]. The leading causes of cirrhosis vary geographically, with endemic hepatitis B (HBV) being the leading cause in Asian pacific countries and hepatitis C (HCV) and alcohol-associated liver disease a major risk factors in Europe, America, Russia, and Australasia [2,3,4]. Whilst the worldwide prevalence of HBV and HCV-induced HCC is declining in line with hepatitis vaccination programs and the elimination of contaminated bloods products, HCC prevalence is increasing in Western Europe, Australasia, and North America [5], mainly attributable to an increase in non-alcoholic fatty liver disease (NAFLD) and non-alcohol steatohepatitis (NASH), reflective of increasing rates of obesity and type 2 diabetes [5,6]. Its presentation with advanced multinodular disease is common, due to synchronous tumour development or early dissemination [3], and the survival is poor. The 5-year survival rate is less than 10% in several European countries, ranging up to 30% in Japan [7], with the number of cases and deaths predicted to rise over the next 20 years [8].

Guidelines for the management of HCC have been standardised utilising clinical prognostic staging systems such as Barcelona Clinical Liver Cancer (BCLC) classification [9], which stratifies patients based on tumour stage and the severity of underlying liver disease. Suitability for treatment depends on tumour burden, location, and performance status (PS), and takes into account age, comorbidities, and patient preference [10]. In brief, patients with asymptomatic very early stage or early-stage disease and preserved liver function are considered for tumour resection, ablation, or transplantation. Trans-arterial chemoembolisation (TACE) can be considered in asymptomatic multifocal intermediate stage disease, and systemic therapies in can be considered in patients with advanced disease with good PS and preserved liver function [9]. Historically, the role of cytotoxics in advanced HCC (aHCC) has been limited, with HCC considered a relatively chemo-refractory tumour and administration of chemotherapy often being complicated by underlying hepatic dysfunction resulting from chronic inflammation and cirrhosis [11]. However, molecularly targeted agents, in particular tyrosine kinase inhibitors targeting pathways in angiogenesis, have improved survival in advanced disease and have become the backbone of systemic therapy [12,13]. Most recently, exciting advances in immunotherapy have resulted in new therapeutic strategies for this challenging cancer.

## 2. Systemic Therapies in aHCC

Cytotoxic chemotherapy has been largely ineffective in aHCC, with high rates of expression of drug resistant genes [14] and poor tolerance of chemotherapy due to underlying liver dysfunction and cirrhosis. No single agent or combination of cytotoxics are widely accepted as standard of care [15].

In 2007, the anti-angiogenic agent sorafenib became the gold standard treatment for HCC after gaining approval from the Food and Drug Administration (FDA) and European Medicines Agency (EMA). Sorafenib, an oral tyrosine kinase inhibitor (TKI) targeting vascular endothelial growth factor receptors (VEGFRs), platelet-derived growth factor receptor (PDGFRs) and Raf, improved median overall survival (mOS) and time to radiological progression (TTP) compared with placebo in patients with aHCC [12,16]. More recently, the multikinase inhibitor lenvatinib has proven to be non-inferior to sorafenib in the first-line advanced setting [17], and the use of these TKI therapies yields a mOS in the region of 12–14 months in patients with previously untreated aHCC [17].

In addition to these first-line options, several second-line therapies have proven efficacy, including the TKIs regorafenib [18] and cabozantinib [19] and the monoclonal antibody ramucirumab in patients with elevated serum alpha fetoprotein (AFP) over 400 ng/mL [20]. Despite these advances, emerging resistance and toxicity remain barriers to long-term survival [21,22,23].

Immunotherapy has revolutionised oncology [24,25,26,27] delivering durable responses for a subset of patients in a broad range of advanced malignancies [28]. The immune system is pivotal in cancer surveillance. Immune evasion through impaired antigen recognition or by fostering an immunosuppressed tumour microenvironment is a recognised hallmark of cancer [29,30]. The immune checkpoint receptors programmed cell death 1 (PD-1) and cytotoxic-T- lymphocyte-associated protein 4 (CTLA-4), have emerged as promising targets for immune modulation. Checkpoint molecules that disrupt interactions between the checkpoint proteins and their ligands, enhance anti-tumour immunity [31,32,33]. Historically there have been concerns about the safety of ICIs in patients with underlying liver impairment, and early clinical trials frequently excluded patients with viral aetiology liver disease. However, recent studies have demonstrated safety and efficacy in this setting, paving the way for significant advances in management of these patients, as detailed below. 

## 3. ICI in HCC

### 3.1. Single-Agent ICI

#### 3.1.1. CTLA-4 Inhibitors

One of the early studies exploring the safety profile of ICIs in individuals with viral aetiology liver disease and aHCC was the phase II study of the CTLA-4 inhibitor tremelimumab. Twenty patients with HCV-associated aHCC and compensated liver disease (Child–Pugh class A or B) received tremelimumab 15 mg/kg every 90 days [34]. A partial response rate (PRR) of 17.6% and disease control rate (DCR) of 76.4% were reported. Treatment-related adverse events (TRAEs) were similar to those reported in non-cirrhotic populations demonstrating tolerability. These reassuring safety data in a group of patients with impaired liver function opened the door to more comprehensive immunotherapy studies. 

#### 3.1.2. PD-1/PD-L1 Inhibitors

The first single-agent ICIs to receive FDA approval in aHCC were the PD-1 inhibitors nivolumab and pembrolizumab, based on the early phase clinical trials CheckMate 040 and KEYNOTE-224, respectively.

CheckMate 040 was a phase I/II dose escalation and expansion study of nivolumab in patients with aHCC, who had or had not previously received sorafenib. The study included patients with both viral and non-viral aetiology HCC [35]. An objective response rate (ORR) of 15–20% was reported, with a median duration of response (mDOR) of 17 months; a 95% confidence interval (CI) 6–24 in the dose escalation phase; and 9.9-month 95% CI 8.3- non-estimable (NE) in the dose expansion phase. Durable responses to treatment were observed regardless of previous treatment with sorafenib or HCC aetiology (viral vs. non-viral). The safety profile of nivolumab was consistent with that observed in other solid tumours with grade 3–4 TRAEs in the region of 21–33%. The inclusion of patients with viral hepatitis did not result in any new safety signals [35]. Eligible patients with Child–Pugh B liver cirrhosis (n = 49) also demonstrated responses (ORR 12%; 95% CI 5–25 and DCR 55%; 95% CI 40–69) and a manageable toxicity profile (Grade 3–4 TRAEs in 24%, leading to discontinuation in 4%) [36]. 

The PD-1 inhibitor pembrolizumab demonstrated an acceptable safety profile in the phase II KEYNOTE-224 study, in which 104 patients with sorafenib-pre-treated aHCC received the ICI. At an extended median follow up (mFU) of 45.1 months (range 4.3–49.3), the primary endpoint of ORR was 18%; 95% CI 11–27, with a mDOR of 21.0 months (range, 3.1–39.5) and a mOS of 13.2 months, and 95% CI 9.7–15.3. Grade 3–4 TRAEs were reported in 25% of patients, of which there were three episodes of grade 3 or above immune-mediated hepatitis. No viral-induced hepatitis flares were reported [37]. 

Pembrolizumab was further explored in a systemic anti-cancer therapy (SACT)-naive population in a cohort of the same study, in which 51 patients with untreated aHCC received pembrolizumab [38]. The ORR was 16%; 95% CI 7–29 with a mDOR of 16 months (range, 3–24+); similar responses were evidenced regardless of prior treatment. The mOS was 17 months and there was a 95% CI 8–23 months and TRAEs grade 3 or above occurred in 16% of patients. 

These phase I–II studies demonstrated the anti-tumour effects of single-agent immunotherapy in aHCC, evidencing durable tumour responses for a proportion of patients and confirming an acceptable toxicity profile. Subsequently, both nivolumab and pembrolizumab were taken forward into phase III randomised control trials (RCTs). A summary of the results of RCTs involving ICIs in aHCC is shown in Table 1.

In CheckMate 459, previously untreated patients with aHCC were randomised to receive either nivolumab or sorafenib [39]. At an extended follow up of 33.6 months, the mOS in the nivolumab arm was 16.4 months; 95% CI 14.0–18.5 vs. 14.8 months; and 95% CI 12.1–17.3 in the sorafenib arm (hazard ratio (HR) 0.85; 95% CI 0.7–1.0; *p* = 0.0522). Despite the numerically greater OS observed with nivolumab, the study’s pre-defined statistical significance criteria were not met and the study concluded that nivolumab was not superior to sorafenib [39]. It was noted that a proportion of patients (21%) in the sorafenib arm subsequently received ICI at progression, and this may have confounded survival results [49]. Nivolumab had the more favourable safety profile, with a lower rate of Grade 3–4 TRAEs (22% nivolumab vs. 49% sorafenib). Nivolumab for the first line treatment of aHCC was voluntarily withdrawn from the US market in response to these results [50].

Single-agent pembrolizumab was investigated in the second-line setting in KEYNOTE-240, in which 413 patients with aHCC who had previously received sorafenib, were randomised to pembrolizumab (200 mg every 3 weeks) or placebo [47]. Again, despite a numerically greater mOS in the ICI arm compared to placebo (mOS 13.9 months; 95% CI 11.6 to 16.0 vs. 10.6 months; 95% CI 8.3 to 13.5, respectively, HR 0.78; 95% CI 0.611 to 0.998; *p* = 0.0238), the results did not meet the pre-specified statistical significance criteria. The authors acknowledged that the mOS for the control arm was better than anticipated, likely because of new effective therapies available on progression. Subgroup analysis identified that patients from Asia appeared to have a trend towards greater benefit from pembrolizumab [51].

Subsequently, pembrolizumab was investigated in a phase III RCT comparing second-line pembrolizumab to best supportive care (BSC) in an Asian population of patients with aHCC in KEYNOTE 394 [48]. In this study, 453 patients, of which approximately 80% had HBV-associated HCC, were randomised to receive pembrolizumab 200 mg or placebo every 3 weeks up to 35 weeks. At a mFU of 33.8 months (range 18.7–49), pembrolizumab significantly improved OS (mOS 14.6 months; 95% CI 12.6–18.0 in the pembrolizumab arm vs. 13.0 months; 95% CI 10.5–15.1 in the placebo arm, HR 0.79; 95% CI 0.63–099, *p* = 0.0180). A greater ORR of 13%; 95% CI 9.1–17.0 was observed in the pembrolizumab arm compared to 1%; 95% CI 0.2–4.6 in the placebo arm (*p* < 0.0001) and there was an increased frequency of grade 3 or above AEs in the pembrolizumab arm (14%) compared to placebo (6%) [48]. Furthermore, the survival benefit from pembrolizumab compared to placebo was confirmed in a pre-planned meta-analysis of data from KEYNOTE-394 and KEYNOTE-240 [52]. The mOS for patients receiving pembrolizumab was 14.2 months; 95% CI 12.8–16.2 compared to 12.5 months; 95% CI 10.2–13.6 for placebo (HR 0.79; 95% CI 0.67–0.93) in the pooled analysis. The results were consistent across subgroups, including cirrhosis aetiology, BCLC stage, and age, supporting the use of single-agent ICI post TKI therapy in this geographical patient group.

Beyond pembrolizumab and nivolumab, a number of other checkpoint inhibitors have been explored as single-agent treatment in aHCC. The anti-programmed cell death ligand 1 (PD-L1) Tislelizumab, was investigated in the phase III RCT RATIONALE-301 [40]. In this study, 674 patients with aHCC received tislelizumab 200 mg IV 3 weekly or sorafenib in the first-line setting. Tislelizumab was non-inferior to sorafenib, reporting a mOS of 15.9 months in the treatment arm vs. 14.1 months in the control arm (HR 1.1; 95% CI 0.92–1.33). Tislelizumab was associated with a higher ORR compared to sorafenib (14% vs. 5%, respectively), and AEs leading to discontinuation were reported at 11% in the tislelizumab arm and 5% in the sorafenib arm. Health-related QoL outcomes were more favourable with tislelizumab compared to sorafenib [53].

Camrelizumab, a PD-1 inhibitor, was investigated in a phase II open label trial performed in a Chinese population of patients with aHCC, of which 83% had HBV infection and all had previously received systemic therapy [54]. Patients received either camrelizumab 3 mg/kg every 2 or 3 weeks. At a mFU of 12.5 months (IQR 5.7–15.5), an ORR of 15% and 95% CI 10.3–20.2 was observed, and the primary end point of OS probability at 6 months was 74.4% with a 95% CI 68.0–79.7. The mOS for the study population was 13.8 months with a 95% CI 11.5–16.6. There was a manageable toxicity profile, with grade 3–4 AE events reported in 22% of patients. Immune-mediated adverse events IMAEs were reported in 83% of patients, with the most common immune event being reactive capillary endothelial proliferation (67%), of which all incidences were grade 1 or 2.

In summary, single-agent ICIs have demonstrated encouraging ORR in the region of 15–20% and a well-tolerated side effect profile, but failed to demonstrate statistically significant superiority to established TKI therapy, leading researchers to explore combination therapies in efforts to improve efficacy. 

### 3.2. Dual Checkpoint Inhibition 

Combining immune checkpoint blockades may have synergistic effects, and has improved PFS and OS compared to single-agent immunotherapy in several solid cancers, albeit at increased risk of toxicity [55,56,57]. Dual immune checkpoint therapy was approved in the US as a second-line treatment for aHCC based on the findings of the aforementioned CheckMate 040 study. This phase I/II study randomised 148 patients with aHCC who had previously received sorafenib to one of three different dosing regimens of ipilimumab and nivolumab [58]. The trial reported an ORR of 31%, with a mDOR of 17 months. The arm receiving ipilimumab 3 mg/kg and nivolumab 1 mg/kg 3 weekly for 4 cycles, followed by nivolumab 240 mg every 2 weeks, demonstrated the numerically longest mOS of 22.2 months [59]. IMAEs were also highest in this arm with 42% of patients experiencing grade 3–4 AE. However, 90% of these AEs resolved within 6 weeks. Although a clinically meaningful ORR of 32%, 95% CI 20–48, and 12-month overall survival rate of 61% were observed with this combination, the lack of patient stratification and a predefined statistical analysis to compare outcomes between arms limits the conclusions regarding the optimal treatment regimen. The FDA approved ipilimumab and nivolumab on the basis that it is safe and effective, with observed durable responses [58]. The phase III study, CheckMate 9DW, is underway to compare the ipilimumab/nivolumab combination with sorafenib or lenvatinib in the first-line treatment of patients with aHCC (NCT 04039607), with results eagerly awaited.

The HIMALAYA phase III RCT compared three different immunotherapy regimens with sorafenib, randomising 1171 patients with aHCC who had not received prior systemic therapy to either STRIDE—a single loading dose of 300 mg tremelimumab (CTLA-4 monoclonal antibody) and regular interval durvalumab (PD-L1 inhibitor), single-agent durvalumab every 4 weeks, four doses of 75 mg of tremelimumab with durvalumab maintenance (closed early) or sorafenib 400 mg twice daily. Recruitment to the single-agent, low-dose tremelimumab arm was closed early after interim analysis did not detect any difference in outcomes compared with durvalumab alone. At a median follow up of approximately 32 months, the mOS in the STRIDE treatment arm, durvalumab arm and sorafenib arm was 16.4 months; 95% CI 14.16–19.58, 16.6 months; 95% CI 14.06–19.12 and 13.8 months; 95% CI 12.25 -16.1 respectively [41], meeting the primary end point demonstrating a 22% reduction in risk of death for patient receiving STRIDE compared to sorafenib (HR 0.78; 95% CI 0.65–0.93, *p* = 0.0035) [41]. Single-agent durvalumab was non-inferior to sorafenib (HR 0.86; 95% CI 0.73–1.03, noninferiority margin 1.08). There was no significant difference in the frequency of grade 3–4 TRAEs between the three arms (51% in STRIDE, 37% in durvalumab, and 40% in sorafenib). STRIDE was superior to sorafenib in this study, and the addition of tremelimumab to durvalumab numerically improved ORR and mDOR compared to single-agent durvalumab (ORR 20% vs. 17% and mDOR 22.3 months vs. 16.8 months, respectively). The combination of CTLA-4 and PD-L1 antibodies demonstrated an acceptable toxicity profile. The FDA and EMA have approved tremelimumab plus durvalumab in first-line treatment of aHCC based on the result of this study.

### 3.3. ICI/VEGF Inhibition

The overexpression of VEGF has been implicated in the development of liver cancer and promotion of angiogenesis in HCC [60]. VEGF-targeted therapies may enhance the efficacy of PD-L1-targeted ICI by reversing VEGF mediated immunosuppression and promoting T cell infiltration into tumours [61], rationalising the combination of ICI with VEGF therapy. 

IMbrave 150 was a landmark study combining VEGF inhibition with an ICI, resulting in improved OS and establishing a new paradigm in the treatment of aHCC. This phase III study recruited 501 untreated patients with aHCC and randomised in a 2:1 ratio to receive the PD-L1 inhibitor atezolizumab 1200 mg in combination with the VEGF inhibitor bevacizumab 15 mg/kg every 3 weeks or sorafenib 400 mg bd [42,62]. At an extended mFU of 15.6 months, durable responses were evidenced, with atezolizumab and bevacizumab significantly improving mOS compared to sorafenib (19.2 months vs. 13.4 months, respectively, HR 0.66; 95% CI 0.52–0.85, *p* = 0.0009). Survival at 18 months was 52% in the treatment arm vs. 40% in the control arm. An ORR of 30% was reported with the IO/VEGF combination compared to 11% with TKI, and the benefit from the combination was consistent across subgroups [42]. This is the longest mOS reported to date for first-line systemic treatment in a phase III study in patients with aHCC. Subsequently, atezolizumab and bevacizumab are now the accepted first-line regimen of choice for eligible patients. Importantly, this study excluded patients with complications due to portal hypertension, including untreated oesophageal varices, moderate ascites or previous episodes of hepatic encephalopathy [42]. Upper gastrointestinal endoscopy was mandatory within 6 months of enrolment onto the study, due to concerns about possible bleeding events with the use of bevacizumab. There were six grade 5 bleeding events in the atezolizumab and bevacizumab treatment arm and one in the sorafenib arm. Of the patients who died from significant bleeding events after receiving atezolizumab and bevacizumab, all had evidence of microvascular invasion, which is associated with portal hypertension and varices, and three had varices present at baseline. These results highlight the importance of caution when considering this combination in patients with an increased risk of bleeding. 

A further study investigating the combination of ICI with VEGF inhibition, this time in an exclusively viral aetiology aHCC cohort from China, is the phase II/III trial ORIENT 32 [43]. In this study, 571 patients with HCV-HCC were randomised to receive sintilimab (a PD-1 inhibitor) plus IBI305 (a bevacizumab biosimilar) or sorafenib in the first-line setting. The median PFS was 4.6 months in the combination treatment arm compared to 2.8 months with sorafenib. In the first interim analysis, sintilimab–IBI305 evidenced significantly longer OS compared to sorafenib (median OS not reached; 95% CI not reached-not reached in the sintilimab–IBI305 arm compared to 10.4 months; 95% CI 8.5–not reached in the sorafenib arm, HR 0.57; 95% CI 0.43–0.75, *p* < 0.0001). An acceptable safety profile was demonstrated with TRAE grade 3 and above reported at 34% in the treatment arm and 36% in the control arm. Bleeding events leading to discontinuation on trial were reported for ten patients receiving the combination versus two receiving sorafenib. In this study endoscopy to assess bleeding risk was not mandated and performed at investigators discretion. Authors felt the ORIENT-32 population was more representative of the clinic population than IMbrave 150, with a higher rate of extrahepatic metastatic disease, higher proportions of patients previously receiving local liver-directed therapy and including a proportion of patients (4%) with Child–Pugh class B liver cirrhosis [43]. 

Cabozantinib is an oral TKI that targets a range of kinases including VEGF, c-MET, and AXL. Cabozantinib has demonstrated benefit in a pretreated aHCC population compared with placebo [19]. COSMIC-312 was a phase III trial that randomised patients with aHCC to receive cabozantinib with or without atezolizumab or sorafenib in the first-line setting [44]. PFS in the TKI/ICI treatment arm was significantly improved compared to the sorafenib control arm (HR 0.63; 99% CI 0.44–0.91, *p* = 0.0012). The median PFS was 6.8 months with cabozantinib and atezolizumab and 4.2 months with sorafenib. However, at a mFU of 13.6 months, there was no significant improvement in mOS with the combination compared to sorafenib (cabozantinib and atezolizumab 15.4 months; 95% CI 13.7–17.7 vs. sorafenib 15.5 months; 95% CI 12.1- NE; HR 0.90; 96% CI 0.69–1.18, *p* = 0.428).

The combination of anti-PD-1 antibody camrelizumab and VEGFR2-targeted TKI rivoceranib was compared with sorafenib in the first line setting in the phase III RCT CARES 310, recruiting a predominantly Asian population (83%, n = 449). A significantly greater mPFS (5.6 months; 95% CI 5.5–6.3 vs. 3.7 months; 95% CI 2.8–3.7, HR 0.52; 95% CI 0.4–0.65, *p* < 0.0001) and OS (22.1 months; 95% CI 18.1–27.2 vs. 15.2 months; 95% CI 13.0–18.5, HR 0.62; 95% CI 0.49–0.80, *p* < 0.0001) was reported for the combination compared to sorafenib, with benefit observed across the majority of subgroups, including the Asian vs. non-Asian population (OS HR 0.66; 95% CI 0.55–0.6 Asian, 0.55; 95% CI 0.29–1.02 non-Asian) [45]. 

Lenvatinib is an oral TKI that has been compared with sorafenib in the first-line treatment of aHCC in the REFLECT trial, demonstrating non-inferiority in overall survival, and a statistically significant improvement in secondary end points including mPFS and ORR (24% lenvatinib versus 9% for sorafenib) [17]. In the phase III study LEAP 002, lenvatinib was used as the standard of care control arm and was combined with pembrolizumab in the treatment arm in the first-line setting, randomising 794 patients with aHCC to receive lenvatinib and pembrolizumab or lenvatinib and placebo [46]. At a mFU of 32.1 months (range 25.8–41.1), the mOS with lenvatinib and pembrolizumab was numerically greater compared to lenvatinib and placebo (21.2 months vs. 19.0 months, respectively, HR 0.840; 95% CI 0.708–0.997, *p* = 0.0227), and at 24 months, 43.7% of the population were alive in the treatment arm compared to 40.0% in the control arm. Despite a greater OS with the combination, the results did not meet the pre-specified statistical significance (one-sided *p* = 0.002 at interim analysis for PFS and 0.0185 for OS at final analysis), and it was concluded that the lenvatinib and pembrolizumab combination was not superior to lenvatinib and placebo. The combination demonstrated a manageable toxicity profile with grade 3 and above TRAEs reported in 63% receiving lenvatinib and pembrolizumab compared to 58% receiving lenvatinib and placebo, and health-related QoL scores were similar between the treatment groups. Although a negative study, the combination of lenvatinib and pembrolizumab achieved impressive survival results, with the longest mOS reported in a first line study of aHCC (21.2 months). It is notable that the control arm (lenvatinib and placebo) yielded a mOS of 19 months, 6 months longer than reported in the REFLECT study, confirming the role for lenvatinib as a standard of care in first-line aHCC, but noting that this population did not include patients with the poor prognostic finding of main vein portal invasion, unlike other studies such as IMbrave 150. 

Table 2 summarises a selection of other early phase studies investigating ICI with targeted therapies. These phase I and II studies investigated PD-1 directed therapy in combination with antiangiogenics ± immune modulating therapy in the first line and second line setting, demonstrating signals of activity with varying response rates and potentially manageable toxicity profiles.

## 4. Intermediate Stage Disease

Around 60% of HCCs are diagnosed at an intermediate stage (BCLC stage B), describing asymptomatic multifocal disease for which liver directed therapies such as percutaneous ablation or TACE are recommended. Liver-directed therapies induce ischaemia, increase immunogenic cell death and stimulate release of antigens and pro-inflammatory cytokines [34,71], enhancing tumour-specific immune responses [72,73]. The immune modulating effects of local therapies in HCC have prompted investigation into combinations of liver-directed therapy and ICI. 

In a study evaluating the combination of tremelimumab and TACE or radiofrequency ablation in 32 patients with pre-treated HCC, an ORR of 26% and DCR of 89% were observed [74]. Tumour responses were also noted outside of the ablated or embolised zone. The combination of tremelimumab and durvalumab, alongside TACE, has also been shown to be safe and feasible in a cohort of 13 patients with aHCC [75]. Several ongoing clinical trials are investigating ICI in combination with or in comparison to TACE (summarised in Table 3).

In addition to combining ICI with liver-directed therapies, there are ongoing studies investigating sequential ICI/VEGF and local treatment in intermediate-stage disease. This has been prompted by the observation that in IMbrave 150, patients with intermediate-stage disease, unsuitable for TACE, responded favourably to the combination with an OS, PFS and ORR of 25.8 months, 12.6 months and 44%, respectively, compared to 17.5 months, 6.5 months and 27% in the more advanced stage population. A proportion of these responding patients were able to proceed to curative treatments, such as RFA or curative TACE, with a reported 30% (n = 30/101) of patients deemed cancer-free after ‘ABC conversion therapy’ [82]. Beyond sequential treatment, it is unknown whether the ICI/VEGF combination is more efficacious than TACE for the upfront treatment of intermediate HCC, and a phase III RCT will compare atezolizumab/bevacizumab against TACE in intermediate-stage disease with a high tumour burden not suitable for transplant [81].

## 5. Evaluation of Predictive Biomarkers for ICI

Studies of single-agent PD-1 inhibitors in aHCC have evidenced response to treatment in a small proportion of patients (ORR 14–18%) [35,38]. Combining ICI with targeted therapy improves response rate. However, in the pivotal trial IMbrave 150, 20% of patients were refractory to the combination treatment [62], highlighting the importance of continuing to search for clinically meaningful biomarkers to guide patient selection and personalise therapy. Predictive biomarkers not only maximise therapeutic benefit, but are pertinent when considering the rising cost associated with immunotherapy use compared to other treatments, which may limit availability in countries where HCC is most prevalent. Several potential biomarkers have been studied to predict the response to immunotherapy in aHCC, including immune cell infiltration, programmed death-ligand 1 (PD-L1) expression, tumour mutational burden (TMB), and microsatellite instability (MSI).

### 5.1. PD-L1 Expression

PD-L1 is a protein that can be expressed on the surface of cancer cells and interacts with the PD-1 receptor on T cells, suppressing immune response and promoting tumour growth. Clinically validated biomarkers such as PD-L1 expression have clinical utility in tumour groups such as lung, breast, and oesophageal cancers [83,84,85,86]. PD-L1 expression is visualised using various IHC assays and quantified by tumour cell expression (TPS) or tumour and surrounding immune cell expression (CPS) [87]. There is known inter-assay variability in the detection of PD-L1 in HCC tumours [88], which complicates biomarker development and definition of a clinically meaningful threshold for PD-L1 positivity.

Tissue collected from 184 patients receiving single-agent nivolumab as part of CheckMate 040 underwent the IHC quantification of PDL1 TPS. PD-L1 expression of ≥1% associated with an improved survival (mOS of 28.1 months; 95% CI 18.2—not reached in the PD-L1 positive cohort versus 16.6 months; 95% CI 14.4–20.2 in the PD-L1 negative (<1%) cohort, *p* = 0.05) [89,90]. However, the authors were keen to highlight that complete and partial responses also occurred in the patients with <1% PD-L1 expression, implying this biomarker alone is insufficient to guide therapy choices [90]. In the ipilimumab/nivolumab combination cohort of CheckMate 040, responses also occurred regardless of PD-L1 expression (although the study was not sufficiently powered to draw conclusions on the significance of PD-L1 expression) [59].

In the phase II trial KEYNOTE-224, 52 patients were assessed for PD-L1 expression, quantifying both TPS and CPS. A CPS score of ≥1% was associated with a higher ORR to pembrolizumab, compared to a CPS score <1% (ORR 32% vs. 20%, *p* = 0.021, respectively) and prolonged PFS (*p* = 0.026). However, when assessing PD-L1 expression in tumour cells alone (TPS), no correlation between response and survival was found [91]. 

A meta-analysis of 11 open label predominantly phase I/II trials of ICI in aHCC revealed significantly higher ORR in PD-L1-positive patients compared to PD-L1 negative patients (26% vs. 18%) [92]. However, long-term survival outcomes could not be evaluated due to the limited follow up in these early phase trials. Again, durable responses to treatment were observed in both PD-L1-positive and PD-L1-negative patients, suggesting that although the expression of PD-L1 confers a higher likelihood of response to treatment, PD-L1 expression alone does not serve as a comprehensive independent biomarker for patient selection. 

Indeed, as phase III RCT biomarker data are reported, the role of PD-L1 expression in aHCC remains unclear. In CheckMate 459, PD-L1 expression did not predict for greater survival benefit in patients receiving nivolumab (PD-L1 ≥1% HR 0.80; 95% CI 0.54–1.17; PD-L1 <1% HR 0.84; 95% CI 0.70–1.01) [49] and the HIMALAYA trial demonstrated the benefit of STRIDE dual immunotherapy over that of sorafenib, regardless of PD-L1 expression [41]. The clinical utility of PD-L1 testing varies greatly between cancer types and treatment settings [87], and for aHCC, further high-quality RCTs evaluating PD-L1 expression are needed to determine whether the assessment of tumour PD-L1 expression can impact on clinical management.

### 5.2. Tumour Mutation Burden (TMB)

Tumour mutation burden (TMB) is defined as the number of DNA mutations per megabase (muts/Mb) in the coding genome of cancer cells, as determined by the next-generation sequencing of tumour DNA. A high TMB is a predictive biomarker for the response to ICI in multiple solid tumours [93]. However, studies in HCC have demonstrated a low TMB (<5 muts/Mb) compared to other tumour groups [94]. Therefore, high TMB is unlikely to be a relevant biomarker for ICI response in the large majority of patients with HCC [95].

### 5.3. Microsatellite Instability (MSI)

Microsatellite instability (MSI) results from deficiencies in the mismatch repair pathway that maintains DNA integrity, repairing DNA base substitutions and frameshift mutations. MSI high tumours are associated with response to ICI in a range of tumours, particularly those that originate from the gastrointestinal tract [96]. However, MSI high tumours are uncommon in HCC, with <3% of HCCs harbouring an MSI status, limiting clinical utility [96,97]. 

The development of predictive biomarkers has been somewhat hampered by the lack of tissue for translational research work, as this is not always mandated for diagnosis in HCC [98]. In addition, many of the tumour tissue samples available are from resected early-stage disease and may not be representative of the more advanced cancer setting. Diagnostic biopsies at all stages of disease should be encouraged and bio-banked to expedite translational research and biomarker development.

### 5.4. Immune Cell Infiltration/Tumour Microenvironment

Tumour-infiltrating lymphocytes (TILs) play an important role in the immune response to cancer [99]. The presence and frequency of TILs is a prognostic and predictive biomarker, correlating with survival and response to ICI in cancers such as melanoma and non-small-cell lung cancer (NSCLC) [100,101,102]. In HCC, flow cytometry of 21 aHCC tumours evidenced that a high frequency of PD-1 high CD8 positive T cells trended towards increased response to ICI [103]. In a further study of 49 aHCC tumour biopsies, the high expression of CD38 on immune cells was also associated with increased response to ICI (43.5% ORR high proportion of CD38+ cells vs. 3.9% low proportion, *p* = 0.019) and improved survival (mPFS 8.21 months vs. 1.64 months, *p* = 0.0065 and mOS 19.06 months vs. 9.59 months, *p* = 0.0295 for high expression of CD38 vs. low expression, respectively) [104].

In a phase II study of second-line ICI in aHCC, bloods samples from 60 patients receiving pembrolizumab were prospectively collected and underwent molecular characterisation. An increased frequency of activated circulating CD8+ T cells associated with response [105] and RNA profiling of tumour demonstrated that responders had increased T cell receptor signalling activation with higher expressions of major histocompatibility complex (MHC) genes. The authors concluded that a subset of patients with an immune-rich tumour microenvironment and increased frequency of circulating CD8+ T cells responded favourably to pembrolizumab.

In combination with TILs frequency, gene expression profiles that reflect key biological pathways involved in T-cell–directed therapies can inform the response to ICI. Molecular and immune analysis of 83 tumour samples collected from patients with aHCC known to have responded to ICI demonstrated higher levels of intratumoural inflammatory signalling at baseline, including upregulated interferon-γ signalling and MHC II-related antigen presentation [106]. The authors identified an 11-gene signature, which predicted response and survival in patients receiving anti-PD-1. The signature was validated in a further cohort of aHCC and a cohort of other solid malignancies, but was not found to be predictive for patients who had tissue collected at initial diagnosis and received TKI systemic therapy prior to ICI, highlighting the need for serial biopsies and the potential impact of systemic therapy on the tumour microenvironment.

In CheckMate 040, tissue collected underwent IHC quantification of tumour-infiltrating T cells expressing CD3 and CD8 and increased frequency demonstrated a non-significant trend towards improved survival. A inflammatory gene signature (consisting of CD274, PD-L1, CD8A, LAG3, and STAT1) was associated with improved ORR (*p* = 0.05) and OS (*p* = 0.01) [90]. Tumour samples from 358 patients with aHCC receiving atezolizumab and bevacizumab in the GO30140 phase Ib or IMbrave 150 phase III trial underwent molecular analysis, identifying that increased IFN γ signalling, active antigen presentation, and low regulatory T cell (Treg)/Effector-T-cell ratio were associated with response [107]. Patients with high Treg infiltration were associated with significant benefit from the atezolizumab and bevacizumab, compared with atezolizumab monotherapy, suggesting that the combination of ICI with anti-angiogenic therapy may help in overcoming severe Treg infiltration as a resistance mechanism to ICI. Pre-existing immunity in baseline tumour samples was associated with better clinical outcomes from the combination.

A further biomarker of interest is B-Catenin (CTNNB1). Mutations in CTNNB1, resulting in upregulation, improved immune evasion, and this has been associated with resistance to ICI in preclinical studies [107]. Alterations in WNT/β-catenin signalling have been associated with lower DCR and shorter survival in patients with aHCC receiving ICI [108]. In a cohort of patients receiving pembrolizumab, somatic mutations in β-catenin were only detected in non-responders [105]. However, in a separate cohort, the mutational status of β-catenin did not predict resistance to therapy, and mutations were detected in the cohorts of both responders and non-responders [109]. These varying results suggest the need for further studies to evaluate if this is a biomarker for unfavourable response to ICI [110].

These studies highlight the complexity and heterogeneity of molecular and immune profiles within HCC. Although it seems likely that the tumour microenvironment will pre-dispose to favourable responses to ICI, the challenges of obtaining sufficient and contemporaneous tissue, molecular characterisation, and validation of initially positive results remains significant. International quality standards for pathology studies and biomarker development should be adopted in attempts to address the significant challenges associated with inter- and intra-tumour variability, the standardisation of tissue collection, and the processing and interpretation of results. Currently, these novel biomarkers remain limited to translational studies, with no impact on routine clinical practice.

### 5.5. Viral Aetiology and Response to ICI

There is significant interest in the aetiology of chronic liver disease associated with HCC and varying response to ICIs. In murine models, HCC associated with nonalcoholic steatohepatitis (NASH) has demonstrated impaired tumour immune surveillance due to an enrichment of exhausted CD8+PD1+ T cells [111]. A meta-analysis including 1656 patients recruited to the phase III RCTs, CheckMate 459, IMbrave 150, and KEYNOTE 240, demonstrated that patients with viral HCC benefited from ICI (HR 0.64; 95%CI 0.48–0.94); however, patients with non-viral-HCC did not derive significant benefits (HR 0.95; 95% CI 0.77–1.11, *p* of interaction 0.03) [111]. The effect of ICIs appeared similar in viral-HCC regardless of viral aetiology (HBV-HCC HR 0.64; 95% CI 0.49–0.83 vs. HCV-HCC HR 0.6; 95% CI 0.47–0.98). In this study, patients with NASH were not differentiated from other non-viral aetiologies, such as alcoholic liver disease, primary biliary cirrhosis, and cryptogenic cirrhosis. A validation cohort of patients receiving mostly single-agent ICI, consisting of 13 patients with non-alcoholic fatty liver disease (NAFLD) and 117 patients with other-aetiology-HCC, was subsequently considered. NALFD-HCC was an independent poor prognostic factor (HR 2.6; 95% CI 1.2–5.6, *p* = 0.017) and in a further validation cohort of 118 patients with HCC, of which 11 had NALFD, NALFD was associated with reduced survival. 

A further meta-analysis incorporating the same three ICI RCTs assessed if this variation in response associated with HCC aetiology was observed with molecular targeted therapies too, incorporating pooled data from a further five phase III RCT assessing TKI/anti-VEGF (n = 2083), and concluded that no differences related to aetiology were observed in the response of patients treated with TKI/anti-VEGF therapies [109]. These results support the hypothesis that aetiology may be associated with response to ICI and a biological rationale for impaired response to ICI in NALFD, related to the tumour microenvironment has been proposed. However, non-viral HCC is a group encompassing a variety of aetiologies beyond NASH alone, with likely heterogenous response to ICI between the differing aetiology. In addition, the validation cohort consisted of patients with advanced, unresectable disease, with Child–Pugh A liver functional reserve, and documented radiologic or clinical diagnosis of cirrhosis whom received predominantly single-agent ICI and further clinical data will require integration to assess response to combination therapies [112]. Regarding the meta-analysis of phase III RCT incorporating ICI in patients with aHCC, the studies were heterogeneous in terms of treatment combination, control arms, and line of therapy. An independent meta-analysis of the phase III studies KEYNOTE 394 and KEYNOTE 240, both placebo-controlled trials of single-agent pembrolizumab in the second-line setting, found consistent benefit from ICI, regardless of viral aetiology [52].

In the setting of phase III RCTs assessing combination treatments, subgroup analysis of COSMIC 312, suggested that patients with HBV-HCC gained the greatest magnitude of benefit from cabozantinib and atezolizumab, compared to HCV-HCC and non-viral HCC (HR for OS 0.53, 1.10, and 1.18, respectively) [44]. Patients with viral HCC had a greater survival benefit from atezolizumab and bevacizumab in IMbrave 150, compared with non-viral HCC patients (HR 0.51, 0.43 and 0.91 respectively) [42,62], supporting the hypothesis that viral-HCC may respond more favourably to ICI. However, the mOS for the non-viral HCC subgroup receiving atezolizumab and bevacizumab in IMbrave 150 was 17 months, comparable to the mOS of 18.1 months observed in the sorafenib control arm. This not only suggests that ICI/VEGF treatment is effective in non-viral HCC, demonstrating a favourable mOS taking in account historic comparators, but also that patients receiving sorafenib demonstrated unexpectedly durable response potentially related to the post-progression use of ICI, which, in turn, will have impacted the mOS hazard ratio for the non-viral HCC subgroup [112].

In contrast to these finding, similar benefit from camrelizumab and rivoceranib was observed regardless of viral aetiology in CARES 310 (HBV vs. HCV vs. non-viral, OS HR 0.53 (95% CI 0.41–0.68), 0.56 (95% CI 0.22–1.45), and 0.65 (95% CI 0.36–1.20), respectively) [45,113] and again in the HIMALAYA trial, both patients with non-viral HCC and HBV-HCC-derived benefit from STRIDE compared to sorafenib (OS HR non-viral-HCC 0.74; 95% CI 0.57–0.95, HBV-HCC 0.64; 95% CI 0.48–0.86), although, notably, this was not the case with HCV-HCC (HCV-HCC 1.06; 95% CI 0.76–1.49) [114].

These data suggest that responses to ICI are observed in both viral and non-viral-aetiology HCC, but the magnitude of benefit may be greatest in viral HCC. The optimal combination and sequencing of therapies for patients with viral versus non-viral HCC remains a complex clinical challenge, and future thoughtfully designed randomised trials, underpinned by biomarker discovery work from ongoing translational studies of patients treated with ICIs, are required. At the moment, there is no confirmatory data supporting the use of aetiology as a factor for selection of ICI vs. TKI-based first-line therapy in patients with aHCC.

### 5.6. ICI in aHCC and Impaired Liver Function

The role of immunotherapy in poorer prognosis Child–Pugh B patients is yet to be well defined, and further clinical trials are required to evaluate the efficacy and safety of these agents in patients with more severe liver cirrhosis and/or poor prognostic factors, such as microvascular invasion [115]. To date, there is a paucity of data available to assess the magnitude of benefit from ICI in patients with Child–Pugh B cirrhosis compared to Child–Pugh A; however, the limited data available do suggest that the presence of Child–Pugh B cirrhosis does not increase the risk of toxicity from ICI [36,116]. Further prospective studies are required to validate the role of immunotherapy in patients with Child–Pugh B cirrhosis, either as monotherapy or in combination regimens, and potentially identify subgroups of this poorer-prognosis patient group that derive benefit. Tislelizumab is currently under investigation in this setting (NCT05622071).

## 6. Selection of First-Line Therapy in aHCC

The increasing numbers of approved first-line therapeutic strategies for aHCC involving ICIs raises debate as to optimal selection of treatment for individual patients. With options of anti-PD-L1/anti-VEGF combinations, anti-PD-L1/anti-CTLA-4 combination, or TKI alone, there are a number of individual risk factors which may be considered when selecting first-line therapy.

Common adverse events associated with VEGF targeted therapies include hypertension, and proteinuria, and rare but severe adverse events are bowel perforations and gastrointestinal bleed [42]. It is recommended that varices should be evaluated through endoscopy and treated prior to commencing bevacizumab. Bevacizumab is contraindicated for patients with severe active cardiovascular disease and significant kidney impairment and careful consideration is required for patients with a higher risk of bleeding. 

ICI can result in IMAEs that require prompt recognition and specialist expert management [117]. The frequency of IMAEs is higher with ICI combinations compared to single-agent anti-PD-L1 [41]. Historically, patients with autoimmune diseases have been excluded from ICI trials, and the limited evidence base suggests that the incidence of IMAEs is higher in patients with pre-existing autoimmune disease compared with those without an autoimmune diagnosis [118]. ICI should be avoided in cases where a flare of an autoimmune disorder may be life-threatening, in autoimmune neurological or neuromuscular disorders, and in patients receiving high doses of immunosuppression for the treatment of autoimmune disease [119]. The role of ICI in recurrent HCC after liver transplants is not well defined, and PDL 1 plays and important role in graft tolerance [120]. 

At present, there are no molecular or clinical factors which have been proven to predict benefit from either ICI/VEGF combination or dual ICI. To receive this combination treatment, patients are required to be performance status 0 to 1, and there remains a clinical unmet meet for therapies that are suitable for patients with poorer performance status and/or have significant co-morbidities. Since there are no currently available data from randomised studies comparing ICI/VEGF combination with dual ICI selecting between these two options is problematic and is largely based on the numerically higher mOS achieved with ICI/VEGF combination compared to dual ICI, albeit in different studies, and more extensive physician experience with ICI/VEGF combination due to earlier approval.

A further intriguing observation arising from the published randomised studies investigating ICI/VEGF combination is the failure of ICI when combined with TKI to deliver statistically significant improvement in mOS compared to TKI alone (COMIC and LEAP 002 studies) in contrast to ICI combined with anti-VEGR antibody (IMbrave 150). Potential explanations include the different outcomes in the comparator arms of the studies (sorafenib vs. lenvatinib), differences in study design which may have influenced continuation of therapy in patients randomised to the comparator arm (IMbrave150 and COSMIC 312—open-label; LEAP 002—blinded), and potential differences in target pathway inhibition with antibody therapy vs. TKI, or a combination of these and other as yet unknown influences.

**Figure 1:**
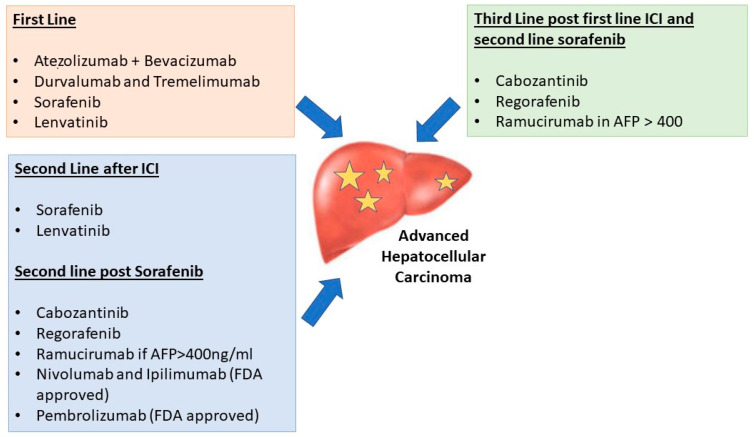
Approved therapies for advanced hepatocellular carcinoma.

Figure 1 provides a summary of approved systemic therapies in advanced HCC.

## 7. Conclusions

The results of IMbrave 150 and HIMALAYA have changed the treatment paradigm for HCC, establishing immune checkpoint inhibition, either combined with anti-angiogenic therapy or dual ICI, as the preferred first-line therapy for eligible patients. In addition, second-line ICI with pembrolizumab or ipilimumab in combination with nivolumab has been approved by the FDA, increasing therapeutic options for some patients diagnosed with this challenging cancer. Undoubtedly, the success of immune checkpoint blockades signals a new era in the management of HCC, with multiple ongoing clinical trials exploring immunotherapy in combinations with established treatments and local-regional therapies. However, a number of remaining questions need to be adequately addressed to allow the unquestionable benefits of ICIs to be optimally leveraged in patients with aHCC. In particular, the sequencing of systemic therapies needs further exploration and definition to maximise tumour responses and the duration of disease control, as patients with aHCC may experience a rapid decline in performance status or liver dysfunction, precluding multiple lines of systemic therapy, and focus should remain on the exploration of clinically impactful prognostic biomarkers to allow identification of patients most likely to benefit from ICI.

## Figures and Tables

**Table 1:** Results of phase III randomised controlled trials involving ICI in aHCC.

Study Drug	Number of Patients	ORR (CR) %	mPFS	mOS (95% CI)	HR	Grade 3–4 TRAE (%)	Primary End Point Met?
**First line**							
Checkmate 459 Yau et al., 2022 [39]							
Nivolumab 240 mg Q2W	371	15 (4)	3.7	16.4 (14.0–18.5)	0.85	22	No—OS did not reach significance per specified criteria
Sorafenib 400 mg BD	372	7 (1)	3.8	14.8 (12.1–17.3)		49	
RATIONALE 301 Qin et al., 2019 [40]							
Tislelizumab 200 mg Q3W	342	14	2.2	15.9 (13.2–19.7)	1.1	11	Yes—OS with tislelizumab non-inferior to sorafenib
Sorafenib 400 mg BD	332	5	3.6	14.1 (12.6–17.4)		5	
HIMALAYA Abou-Alfa et al., 2022 [41]							
STRIDE single loading dose of 300 mg tremelimumab and durvalumab 500 mg Q4W	393	20 (3)	3.78	16.4 (14.1–19.58)	0.78 (STRIDE compared to Sorafenib)	51	Yes—STRIDE significantly improved OS versus sorafenib. Durvalumab monotherapy was noninferior to sorafenib
Single-agent Durvalumab 1500 mg Q4W	389	17 (2)	3.65	16.6 (14.1–19.1)	0.86 (non-inferior to Sorafenib)	37	
Sorafenib	389	20 (0)	4.07	13.8 (12.3–16.1)		40	
IMBRAVE 150 Cheng et al., 2022 [42]							
Atezolizumab 1200 mg, Q3W plus bevacizumab 5 mg/kg Q3W	336	30 (8)	6.9	19.2 (17.0–23.7)	0.66	43	Yes—atezolizumab combined with bevacizumab resulted in better OS and PFS than sorafenib
Sorafenib 400 mg BD	165	11 (<1)	4.3	13.4 (11.4–16.9)		46	
ORIENT 32 Ren at al., 2021 [43]							
Sintilimab 200 mg Q3W plus IBI305 15 mg/kg Q3W	380	20 (1)	4.6	NE (NE-NE)	0.57	34	Yes—sintilimab plus IBI305 showed a significant OS and PFS benefit versus sorafenib
Sorafenib 400 mg BD	191	5 (0)	2.8	10.4 (8.5-NR)		36	
COSMIC 312 Kelley et al., 2022 [44]							
Cabozantinib 40 mg OD and atezolizumab 1200 mg Q3W	432	11 (1)	6.8	15.4 (13.7–17.7)	0.90 (compared with Sorafenib)	64	In part—primary PFS was significantly longer in the combination treatment group versus the sorafenib group. At interim analysis OS did not differ significantly between the treatment groups
Cabozantinib 50 mg OD	118	6 (0)	5.8			46	
Sorafenib 400 mg BD	217	4 (0)	4.2	15.5 (12.1-NE)		60	
CARES 310 Qin, Chan, et al., 2023 [45]							
Camreliziumab 200 mg Q2W and rivoceranib 250 mg PO QDS	272	25 (1)	5.6	22.1 (19.1–27.2)	0.62	81	Yes—camrelizumab and rivoceranib significantly prolonged PFS and OS and improved ORR versus sorafenib
Sorafenib 400 mg BD	271	6 (0.4)	3.7	15.2 (13.0–18.5)		52	
LEAP 002 Finn et al., 2022 [46]							
Lenvatinib 8 mg or 12 mg OD plus pembrolizumab 200 mg Q3W	395	26	8.2	21.2	0.84	63	No—OS and PFS did not meet pre-specified statistical significance
Lenvatinib 8 mg of 12 mg OD plus placebo	399	17	8.1	19.0		58	
**Second Line**							
Keynote 240 Finn et al., 2020 [47]							
Pembrolizumab 300 mg, Q3W	278	18 (2)	3.0	13.9 (11.6–16.0)	0.78	53	No—OS and PFS did not reach significance per specified criteria
Placebo	135	4 (0)	2.8	10.6 (8.3–13.5)		46	
Keynote 394 Qin, Chen, et al., 2022 [48]							
Pembrolizumab 200 mg Q2W	300	13	2.9	14.6 (12.6–18.0)	0.79	14	Yes—pembrolizumab did significantly improve OS, PFS and ORR
Placebo	153	1	2.3	13.0 (10.5–15.1)		6	

Abbreviations: ORR, objective response rate; CR, complete response; mPFS, median progression-free survival; mOS, median overall survival; CI, confidence interval; HR, hazard ratio; TRAE, treatment-related adverse event.

**Table 2:** Selected non-randomised trials exploring ICI in combination with targeted therapies in aHCC.

Trial Name/ID	Phase	Regimen	Targets	Indication	N	Primary Endpoint	ORR	Grade 3–4 TRAE
NCT04444167 Bai et al., 2021 [63]	I/II	AK104 (IV 6 mg/kg Q2W) and Lenvatinib	PD-1/CTLA-4 and VEGF	First-line	18	ORR	ORR 44.4% DCR 77.8%	26.7%
NCT03519997 Hsieh et al., 2023 [64]	II	Pembrolizumab (IV 200 mg Q3W) and Bavituximab (IV 3 mg/kg weekly)	PD-1 and anti-phosphatidylserine	First-line	28	ORR	ORR 32% DCR 61%	Not reported
RENOBATE Yoo et al., 2022 [65]	II	Nivolumab (IV 480 mg Q4W) and Regorafenib (po 80 mg daily for 21 consecutive days Q4W)	PD-1 and VEGF	First-line	42	ORR	ORR 31.0%.	Not reported
NCT03941873 Zhang et al., 2022 [66]	I	Tislelizumab (IV 200 mg Q3W) and Sitravatinib (80 mg/120 mg daily)	PD-1 and VEGF	First or later lines	43	Safety	ORR 10.0%. DCR 85.0%	48.8%
IMMUNIB Vogel et al., 2022 [67]	II	Nivolumab (IV 240 MG Q2W up to 36 cycles) and Lenvatinib	PD-1 and VEGF	First-line	50	ORR	ORR 28%	59.1%
GOING Sanduzzi Zamparelli et al., 2022 [68]	I/II	Nivolumab (1.5 mg/kg, 3 mg/kg or 240 mg Q2W) and Regorafenib (160 mg/day 3W on 1W off in the first 8W)	PD-1 and VEGF	Second-line	51	Safety	Not reported	Less than one third of the patients
Liver100 Kudo et al., 2021 [69]	Ib	Avelumab 10 mg/kg intravenously every 2 weeks plus Axitinib 5 mg orally twice daily	PD-1 and VEGF	First-line	22	Safety and ORR	ORR 13.6%	72.7%
CheckMate 040 Yau et al., 2023 [70]	I/II	Nivolumab 240 mg once every 2 weeks plus Cabozantinib 40 mg once daily (doublet arm); or Nivolumab 3 mg/kg every 2 weeks plus Cabozantinib 40 mg once daily with Ipilimumab 1 mg/kg once every 6 weeks (triplet arm).	PD-1± CTLA4 and VEGF	First- or second-line	71	Safety ORR	ORR Doublet 17% Triplet 29%	Doublet 50% Triplet 74%

Abbreviations: ORR, objective response rate; DCR, disease control rate.

**Table 3:** Ongoing studies combining ICI with TACE, or comparing ICI with TACE in the treatment of intermediate stage HCC.

Study Name	Study Population (n)	Drug	Trial No/Reference
EMERALD 1	710	TACE + Durvalumab + Bevacizumab vs. TACE + Durvalumab + placebo vs. TACE + placebo + placebo	NCT03778957 Sangro, Kudo, et al., 2020 [76]
CHECKMATE 74W	765	TACE + Nivolumab + Ipilimumab vs. TACE + Nivolumab + placebo vs. TACE + placebo + placebo	NCT04340193 Sangro et al., 2021 [77]
LEAP 012	950	TACE + Pembrolizumab + Lenvatinib vs. TACE + placebo + placebo	NCT04246177 Llovet et al., 2022 [78]
TACE 3	522	Drug eluting bead TACE + Nivolumab vs. drug-eluting bead TACE	NCT04268888 Kloeckner et al., 2021 [79]
TALENTACE	342	On-demand TACE combined with Atezolizumab + Bevacizumab vs. on-demand TACE	NCT04712643 Kudo, Guo, et al., 2022 [80]
ABC HCC	434	Atezolizumab plus Bevacizumab vs. TACE	NCT04803994 Foerster et al., 2022 [81]
RENOTACE	496	Regorafenib and Nivolumab vs. TACE	NCT04777851

Abbreviations: TACE, trans-arterial chemoembolisation.

## References

[1] Sung H., Ferlay J., Siegel R.L., Laversanne M., Soerjomataram I., Jemal A., Bray F. (2021). Global Cancer Statistics 2020: GLOBOCAN Estimates of Incidence and Mortality Worldwide for 36 Cancers in 185 Countries. CA Cancer J. Clin..

[2] McGlynn K.A., Petrick J.L., El-Serag H.B. (2021). Epidemiology of Hepatocellular Carcinoma. Hepatology.

[3] Llovet J.M., Zucman-Rossi J., Pikarsky E., Sangro B., Schwartz M., Sherman M., Gores G. (2016). Hepatocellular Carcinoma. Nat. Rev. Dis. Primers.

[4] Huang D.Q., Terrault N.A., Tacke F., Gluud L.L., Arrese M., Bugianesi E., Loomba R. (2023). Global Epidemiology of Cirrhosis—Aetiology, Trends and Predictions. Nat. Rev. Gastroenterol. Hepatol..

[5] Dasgupta P., Henshaw C., Youlden D.R., Clark P.J., Aitken J.F., Baade P.D. (2020). Global Trends in Incidence Rates of Primary Adult Liver Cancers: A Systematic Review and Meta-Analysis. Front. Oncol..

[6] Llovet J.M., Kelley R.K., Villanueva A., Singal A.G., Pikarsky E., Roayaie S., Lencioni R., Koike K., Zucman-Rossi J., Finn R.S. (2021). Hepatocellular Carcinoma. Nat. Rev. Dis. Primers.

[7] Allemani C., Matsuda T., Di Carlo V., Harewood R., Matz M., Nikšić M., Bonaventure A., Valkov M., Johnson C.J., Estève J. (2018). Global Surveillance of Trends in Cancer Survival 2000-14 (CONCORD-3): Analysis of Individual Records for 37 513 025 Patients Diagnosed with One of 18 Cancers from 322 Population-Based Registries in 71 Countries. Lancet.

[8] Rumgay H., Arnold M., Ferlay J., Lesi O., Cabasag C.J., Vignat J., Laversanne M., McGlynn K.A., Soerjomataram I. (2022). Global Burden of Primary Liver Cancer in 2020 and Predictions to 2040. J. Hepatol..

[9] Llovet J.M., Brú C., Bruix J. (1999). Prognosis of Hepatocellular Carcinoma: The BCLC Staging Classification. Semin. Liver Dis..

[10] Galle P.R., Forner A., Llovet J.M., Mazzaferro V., Piscaglia F., Raoul J.-L., Schirmacher P., Vilgrain V. (2018). EASL Clinical Practice Guidelines: Management of Hepatocellular Carcinoma. J. Hepatol..

[11] Lohitesh K., Chowdhury R., Mukherjee S. (2018). Resistance a Major Hindrance to Chemotherapy in Hepatocellular Carcinoma: An Insight. Cancer Cell Int..

[12] Llovet J.M., Ricci S., Mazzaferro V., Hilgard P., Gane E., Blanc J.-F., de Oliveira A.C., Santoro A., Raoul J.-L., Forner A. (2008). Sorafenib in Advanced Hepatocellular Carcinoma. N. Engl. J. Med..

[13] da Fonseca L.G., Reig M., Bruix J. (2020). Tyrosine Kinase Inhibitors and Hepatocellular Carcinoma. Clin. Liver Dis..

[14] Marin J.J.G., Macias R.I.R., Monte M.J., Romero M.R., Asensio M., Sanchez-Martin A., Cives-Losada C., Temprano A.G., Espinosa-Escudero R., Reviejo M. (2020). Molecular Bases of Drug Resistance in Hepatocellular Carcinoma. Cancers.

[15] Zhu A.X. (2006). Systemic Therapy of Advanced Hepatocellular Carcinoma: How Hopeful Should We Be?. Oncologist.

[16] Cheng A.-L., Guan Z., Chen Z., Tsao C.-J., Qin S., Kim J.S., Yang T.-S., Tak W.Y., Pan H., Yu S. (2012). Efficacy and Safety of Sorafenib in Patients with Advanced Hepatocellular Carcinoma According to Baseline Status: Subset Analyses of the Phase III Sorafenib Asia–Pacific Trial. Eur. J. Cancer.

[17] Kudo M., Finn R.S., Qin S., Han K.-H., Ikeda K., Piscaglia F., Baron A., Park J.-W., Han G., Jassem J. (2018). Lenvatinib versus Sorafenib in First-Line Treatment of Patients with Unresectable Hepatocellular Carcinoma: A Randomised Phase 3 Non-Inferiority Trial. Lancet.

[18] Bruix J., Qin S., Merle P., Granito A., Huang Y.-H., Bodoky G., Pracht M., Yokosuka O., Rosmorduc O., Breder V. (2017). Regorafenib for Patients with Hepatocellular Carcinoma Who Progressed on Sorafenib Treatment (RESORCE): A Randomised, Double-Blind, Placebo-Controlled, Phase 3 Trial. Lancet.

[19] Abou-Alfa G.K., Meyer T., Cheng A.-L., El-Khoueiry A.B., Rimassa L., Ryoo B.-Y., Cicin I., Merle P., Chen Y., Park J.-W. (2018). Cabozantinib in Patients with Advanced and Progressing Hepatocellular Carcinoma. N. Engl. J. Med..

[20] Zhu A.X., Kang Y.-K., Yen C.-J., Finn R.S., Galle P.R., Llovet J.M., Assenat E., Brandi G., Pracht M., Lim H.Y. (2019). Ramucirumab after Sorafenib in Patients with Advanced Hepatocellular Carcinoma and Increased α-Fetoprotein Concentrations (REACH-2): A Randomised, Double-Blind, Placebo-Controlled, Phase 3 Trial. Lancet Oncol..

[21] Tian Y., Lei Y., Fu Y., Sun H., Wang J., Xia F. (2022). Molecular Mechanisms of Resistance to Tyrosine Kinase Inhibitors Associated with Hepatocellular Carcinoma. Curr. Cancer Drug Targets.

[22] Zhai B. (2013). Mechanisms of Resistance to Sorafenib and the Corresponding Strategies in Hepatocellular Carcinoma. World J. Hepatol..

[23] Rimassa L., Danesi R., Pressiani T., Merle P. (2019). Management of Adverse Events Associated with Tyrosine Kinase Inhibitors: Improving Outcomes for Patients with Hepatocellular Carcinoma. Cancer Treat. Rev..

[24] Rathmell W.K., Rumble R.B., Van Veldhuizen P.J., Al-Ahmadie H., Emamekhoo H., Hauke R.J., Louie A.V., Milowsky M.I., Molina A.M., Rose T.L. (2022). Management of Metastatic Clear Cell Renal Cell Carcinoma: ASCO Guideline. J. Clin. Oncol..

[25] Govindan R., Aggarwal C., Antonia S.J., Davies M., Dubinett S.M., Ferris A., Forde P.M., Garon E.B., Goldberg S.B., Hassan R. (2022). Society for Immunotherapy of Cancer (SITC) Clinical Practice Guideline on Immunotherapy for the Treatment of Lung Cancer and Mesothelioma. J. Immunother. Cancer.

[26] Robert C. (2020). A Decade of Immune-Checkpoint Inhibitors in Cancer Therapy. Nat. Commun..

[27] Switzer B., Puzanov I., Skitzki J.J., Hamad L., Ernstoff M.S. (2022). Managing Metastatic Melanoma in 2022: A Clinical Review. JCO Oncol. Pract..

[28] Borcoman E., Kanjanapan Y., Champiat S., Kato S., Servois V., Kurzrock R., Goel S., Bedard P., Le Tourneau C. (2019). Novel Patterns of Response under Immunotherapy. Ann. Oncol..

[29] Rabinovich G.A., Gabrilovich D., Sotomayor E.M. (2007). Immunosuppressive Strategies That Are Mediated by Tumor Cells. Annu. Rev. Immunol..

[30] Hanahan D., Weinberg R.A. (2011). Hallmarks of Cancer: The Next Generation. Cell.

[31] Pardoll D.M. (2012). The Blockade of Immune Checkpoints in Cancer Immunotherapy. Nat. Rev. Cancer.

[32] Robinson M.W., Harmon C., O’Farrelly C. (2016). Liver Immunology and Its Role in Inflammation and Homeostasis. Cell. Mol. Immunol..

[33] Keenan B.P., Fong L., Kelley R.K. (2019). Immunotherapy in Hepatocellular Carcinoma: The Complex Interface between Inflammation, Fibrosis, and the Immune Response. J. Immunother. Cancer.

[34] Sangro B., Gomez-Martin C., de la Mata M., Iñarrairaegui M., Garralda E., Barrera P., Riezu-Boj J.I., Larrea E., Alfaro C., Sarobe P. (2013). A Clinical Trial of CTLA-4 Blockade with Tremelimumab in Patients with Hepatocellular Carcinoma and Chronic Hepatitis C. J. Hepatol..

[35] El-Khoueiry A.B., Sangro B., Yau T., Crocenzi T.S., Kudo M., Hsu C., Kim T.-Y., Choo S.-P., Trojan J., Welling T.H. (2017). Nivolumab in Patients with Advanced Hepatocellular Carcinoma (CheckMate 040): An Open-Label, Non-Comparative, Phase 1/2 Dose Escalation and Expansion Trial. Lancet.

[36] Kudo M., Matilla A., Santoro A., Melero I., Gracián A.C., Acosta-Rivera M., Choo S.-P., El-Khoueiry A.B., Kuromatsu R., El-Rayes B. (2021). CheckMate 040 Cohort 5: A Phase I/II Study of Nivolumab in Patients with Advanced Hepatocellular Carcinoma and Child-Pugh B Cirrhosis. J. Hepatol..

[37] Kudo M., Finn R.S., Edeline J., Cattan S., Ogasawara S., Palmer D.H., Verslype C., Zagonel V., Fartoux L., Vogel A. (2022). Updated Efficacy and Safety of KEYNOTE-224: A Phase II Study of Pembrolizumab in Patients with Advanced Hepatocellular Carcinoma Previously Treated with Sorafenib. Eur. J. Cancer.

[38] Verset G., Borbath I., Karwal M., Verslype C., Van Vlierberghe H., Kardosh A., Zagonel V., Stal P., Sarker D., Palmer D.H. (2022). Pembrolizumab Monotherapy for Previously Untreated Advanced Hepatocellular Carcinoma: Data from the Open-Label, Phase II KEYNOTE-224 Trial. Clin. Cancer Res..

[39] Yau T., Park J.-W., Finn R.S., Cheng A.-L., Mathurin P., Edeline J., Kudo M., Harding J.J., Merle P., Rosmorduc O. (2022). Nivolumab versus Sorafenib in Advanced Hepatocellular Carcinoma (CheckMate 459): A Randomised, Multicentre, Open-Label, Phase 3 Trial. Lancet Oncol..

[40] Qin S., Finn R.S., Kudo M., Meyer T., Vogel A., Ducreux M., Macarulla T.M., Tomasello G., Boisserie F., Hou J. (2019). RATIONALE 301 Study: Tislelizumab versus Sorafenib as First-Line Treatment for Unresectable Hepatocellular Carcinoma. Future Oncol..

[41] Abou-Alfa G.K., Chan S.L., Kudo M., Lau G., Kelley R.K., Furuse J., Sukeepaisarnjaroen W., Kang Y.-K., Dao T.V., De Toni E.N. (2022). Phase 3 Randomized, Open-Label, Multicenter Study of Tremelimumab (T) and Durvalumab (D) as First-Line Therapy in Patients (Pts) with Unresectable Hepatocellular Carcinoma (UHCC): HIMALAYA. J. Clin. Oncol..

[42] Cheng A.-L., Qin S., Ikeda M., Galle P.R., Ducreux M., Kim T.-Y., Lim H.Y., Kudo M., Breder V., Merle P. (2022). Updated Efficacy and Safety Data from IMbrave150: Atezolizumab plus Bevacizumab vs. Sorafenib for Unresectable Hepatocellular Carcinoma. J. Hepatol..

[43] Ren Z., Xu J., Bai Y., Xu A., Cang S., Du C., Li Q., Lu Y., Chen Y., Guo Y. (2021). Sintilimab plus a Bevacizumab Biosimilar (IBI305) versus Sorafenib in Unresectable Hepatocellular Carcinoma (ORIENT-32): A Randomised, Open-Label, Phase 2-3 Study. Lancet Oncol..

[44] Kelley R.K., Rimassa L., Cheng A.-L., Kaseb A., Qin S., Zhu A.X., Chan S.L., Melkadze T., Sukeepaisarnjaroen W., Breder V. (2022). Cabozantinib plus Atezolizumab versus Sorafenib for Advanced Hepatocellular Carcinoma (COSMIC-312): A Multicentre, Open-Label, Randomised, Phase 3 Trial. Lancet Oncol..

[45] Qin S., Chan S.L., Gu S., Bai Y., Ren Z., Lin X., Chen Z., Jia W., Jin Y., Guo Y. (2023). Camrelizumab plus Rivoceranib versus Sorafenib as First-Line Therapy for Unresectable Hepatocellular Carcinoma (CARES-310): A Randomised, Open-Label, International Phase 3 Study. Lancet.

[46] Finn R.S., Kudo M., Merle P., Meyer T., Qin S., Ikeda M., Xu R., Edeline J., Ryoo B.-Y., Ren Z. (2022). LBA34 Primary Results from the Phase III LEAP-002 Study: Lenvatinib plus Pembrolizumab versus Lenvatinib as First-Line (1L) Therapy for Advanced Hepatocellular Carcinoma (AHCC). Ann. Oncol..

[47] Finn R.S., Ryoo B.-Y., Merle P., Kudo M., Bouattour M., Lim H.Y., Breder V., Edeline J., Chao Y., Ogasawara S. (2020). Pembrolizumab As Second-Line Therapy in Patients with Advanced Hepatocellular Carcinoma in KEYNOTE-240: A Randomized, Double-Blind, Phase III Trial. J. Clin. Oncol..

[48] Qin S., Chen Z., Fang W., Ren Z., Xu R., Ryoo B.-Y., Meng Z., Bai Y., Chen X., Liu X. (2022). Pembrolizumab plus Best Supportive Care versus Placebo plus Best Supportive Care as Second-Line Therapy in Patients in Asia with Advanced Hepatocellular Carcinoma (HCC): Phase 3 KEYNOTE-394 Study. J. Clin. Oncol..

[49] Sangro B., Park J., Finn R., Cheng A., Mathurin P., Edeline J., Kudo M., Han K., Harding J., Merle P. (2020). LBA-3 CheckMate 459: Long-Term (Minimum Follow-up 33.6 Months) Survival Outcomes with Nivolumab versus Sorafenib as First-Line Treatment in Patients with Advanced Hepatocellular Carcinoma. Ann. Oncol..

[50] Squibb BM Bristol Myers Squibb Statement on Opdivo® (Nivolumab) Monotherapy Post-Sorafenib Hepatocellular Carcinoma U.S. Indication 2021. https://news.bms.com/news/corporate-financial/2021/Bristol-Myers-Squibb-Statement-on-Opdivo-nivolumab-Monotherapy-Post-Sorafenib-Hepatocellular-Carcinoma-U.S.-Indication/default.aspx.

[51] Kudo M., Lim H.Y., Cheng A.-L., Chao Y., Yau T., Ogasawara S., Kurosaki M., Morimoto N., Ohkawa K., Yamashita T. (2021). Pembrolizumab as Second-Line Therapy for Advanced Hepatocellular Carcinoma: A Subgroup Analysis of Asian Patients in the Phase 3 KEYNOTE-240 Trial. Liver Cancer.

[52] Finn R.S., Gu K., Chen X., Merle P., Lee K.-H., Bouattour M., Cao P., Wang W., Cheng A.-L., Zhu L. (2022). Abstract CT222: Pembrolizumab (Pembro) for Previously Treated Advanced Hepatocellular Carcinoma (AHCC): Meta-Analysis of the Phase 3 KEYNOTE-240 and KEYNOTE-394 Studies. Cancer Res..

[53] Finn R.S., Qin S., Kudo M., Meyer T., Boisserie F., Li S., Chen Y., Barnes G., Abdrashitov R., Zhu A.X. (2023). Tislelizumab versus Sorafenib in First-Line Treatment of Unresectable Hepatocellular Carcinoma: Impact on Health-Related Quality of Life in RATIONALE-301 Population. J. Clin. Oncol..

[54] Qin S., Ren Z., Meng Z., Chen Z., Chai X., Xiong J., Bai Y., Yang L., Zhu H., Fang W. (2020). Camrelizumab in Patients with Previously Treated Advanced Hepatocellular Carcinoma: A Multicentre, Open-Label, Parallel-Group, Randomised, Phase 2 Trial. Lancet Oncol..

[55] Larkin J., Chiarion-Sileni V., Gonzalez R., Grob J.-J., Rutkowski P., Lao C.D., Cowey C.L., Schadendorf D., Wagstaff J., Dummer R. (2019). Five-Year Survival with Combined Nivolumab and Ipilimumab in Advanced Melanoma. N. Engl. J. Med..

[56] Motzer R.J., Tannir N.M., McDermott D.F., Arén Frontera O., Melichar B., Choueiri T.K., Plimack E.R., Barthélémy P., Porta C., George S. (2018). Nivolumab plus Ipilimumab versus Sunitinib in Advanced Renal-Cell Carcinoma. N. Engl. J. Med..

[57] Hellmann M.D., Paz-Ares L., Bernabe Caro R., Zurawski B., Kim S.-W., Carcereny Costa E., Park K., Alexandru A., Lupinacci L., de la Mora Jimenez E. (2019). Nivolumab plus Ipilimumab in Advanced Non–Small-Cell Lung Cancer. N. Engl. J. Med..

[58] El-Khoueiry A.B., Yau T., Kang Y.-K., Kim T.-Y., Santoro A., Sangro B., Melero I., Kudo M., Hou M.-M., Matilla A. (2021). Nivolumab (NIVO) plus Ipilimumab (IPI) Combination Therapy in Patients (Pts) with Advanced Hepatocellular Carcinoma (AHCC): Long-Term Results from CheckMate 040. J. Clin. Oncol..

[59] Yau T., Kang Y.-K., Kim T.-Y., El-Khoueiry A.B., Santoro A., Sangro B., Melero I., Kudo M., Hou M.-M., Matilla A. (2020). Efficacy and Safety of Nivolumab Plus Ipilimumab in Patients with Advanced Hepatocellular Carcinoma Previously Treated with Sorafenib. JAMA Oncol..

[60] Morse M.A., Sun W., Kim R., He A.R., Abada P.B., Mynderse M., Finn R.S. (2019). The Role of Angiogenesis in Hepatocellular Carcinoma. Clin. Cancer Res..

[61] Hegde P.S., Wallin J.J., Mancao C. (2018). Predictive Markers of Anti-VEGF and Emerging Role of Angiogenesis Inhibitors as Immunotherapeutics. Semin. Cancer Biol..

[62] Finn R.S., Qin S., Ikeda M., Galle P.R., Ducreux M., Kim T.-Y., Kudo M., Breder V., Merle P., Kaseb A.O. (2020). Atezolizumab plus Bevacizumab in Unresectable Hepatocellular Carcinoma. N. Engl. J. Med..

[63] Bai L., Sun M., Xu A., Bai Y., Wu J., Shao G., Song L., Jin X., Song W., Li B. (2021). Phase 2 Study of AK104 (PD-1/CTLA-4 Bispecific Antibody) plus Lenvatinib as First-Line Treatment of Unresectable Hepatocellular Carcinoma. J. Clin. Oncol..

[64] Hsieh D., Kainthla R., Zhu H., Beg M.S. (2023). Phase 2 Trial of Pembrolizumab (Pembro) and Bavituximab (Bavi) in Advanced Hepatocellular Carcinoma (HCC). J. Clin. Oncol..

[65] Yoo C., Ryoo B.-Y., Kim H.-D., Ryu M.-H., Kang B., Chon H.J., Hong J.Y., Lim H.Y. (2022). Regorafenib plus Nivolumab as First-Line Therapy for Unresectable Hepatocellular Carcinoma (UHCC): Multicenter Phase 2 Trial (RENOBATE). J. Clin. Oncol..

[66] Zhang F., Bai Y., Fang W., Meng Z., Xiong J., Guo Y., Zhang T., Zhang J., Ying J., Chen Z. (2022). Safety, Tolerability, and Preliminary Antitumor Activity of Sitravatinib plus Tislelizumab (TIS) in Patients (Pts) with Unresectable Locally Advanced or Metastatic Hepatocellular Carcinoma (HCC). J. Clin. Oncol..

[67] Vogel A., Siegler G.M., Siebler J., Lindig U., Schultheiß M., Müller T., Simon H., Jöckel C., Mueller D.W., Al-Batran S.-E. (2022). IMMUNIB Trial (AIO-HEP-0218/Ass): A Single-Arm, Phase II Study Evaluating Safety and Efficacy of Immunotherapy Nivolumab in Combination with Lenvatinib in Advanced-Stage Hepatocellular Carcinoma (HCC). J. Clin. Oncol..

[68] Sanduzzi Zamparelli M., Matilla A., Lledó J.L., Martínez S.M., Varela M., Iñarrairaegui M., Perelló C., Minguez B., Llarch N., Márquez L. (2022). Early Nivolumab Addition to Regorafenib in Patients with Hepatocellular Carcinoma Progressing under First-Line Therapy (GOING Trial), Interim Analysis and Safety Profile. J. Clin. Oncol..

[69] Kudo M., Motomura K., Wada Y., Inaba Y., Sakamoto Y., Kurosaki M., Umeyama Y., Kamei Y., Yoshimitsu J., Fujii Y. (2021). Avelumab in Combination with Axitinib as First-Line Treatment in Patients with Advanced Hepatocellular Carcinoma: Results from the Phase 1b VEGF Liver 100 Trial. Liver Cancer.

[70] Yau T., Zagonel V., Santoro A., Acosta-Rivera M., Choo S.P., Matilla A., He A.R., Cubillo Gracian A., El-Khoueiry A.B., Sangro B. (2023). Nivolumab Plus Cabozantinib With or Without Ipilimumab for Advanced Hepatocellular Carcinoma: Results from Cohort 6 of the CheckMate 040 Trial. J. Clin. Oncol..

[71] Guo L.Y., Zhu P., Jin X.P. (2016). Association between the Expression of HIF-1α and VEGF and Prognostic Implications in Primary Liver Cancer. Genet. Mol. Res..

[72] Mizukoshi E., Yamashita T., Arai K., Sunagozaka H., Ueda T., Arihara F., Kagaya T., Yamashita T., Fushimi K., Kaneko S. (2013). Enhancement of Tumor-Associated Antigen-Specific T Cell Responses by Radiofrequency Ablation of Hepatocellular Carcinoma. Hepatology.

[73] Leuchte K., Staib E., Thelen M., Gödel P., Lechner A., Zentis P., Garcia-Marquez M., Waldschmidt D., Datta R.R., Wahba R. (2021). Microwave Ablation Enhances Tumor-Specific Immune Response in Patients with Hepatocellular Carcinoma. Cancer Immunol. Immunother..

[74] Duffy A.G., Ulahannan S.V., Makorova-Rusher O., Rahma O., Wedemeyer H., Pratt D., Davis J.L., Hughes M.S., Heller T., ElGindi M. (2017). Tremelimumab in Combination with Ablation in Patients with Advanced Hepatocellular Carcinoma. J. Hepatol..

[75] Buchalter J., Browne I., Mac Eochagain C., Flynn C., Carroll H.K., Galligan M., Bourke M., Lester-Grant A., Desmond F., Hoey K. (2022). Tremelimumab (Day 1 Only) and Durvalumab in Combination with Transarterial Chemoemobilization (TACE) in Patients with Hepatocellular Carcinoma (HCC). J. Clin. Oncol..

[76] Sangro B., Kudo M., Qin S., Ren Z., Chan S., Joseph E., Arai Y., Mann H., Morgan S., Cohen G. (2020). P-347 A Phase 3, Randomized, Double-Blind, Placebo-Controlled Study of Transarterial Chemoembolization Combined with Durvalumab or Durvalumab plus Bevacizumab Therapy in Patients with Locoregional Hepatocellular Carcinoma: EMERALD-1. Ann. Oncol..

[77] Sangro B., Harding J.J., Johnson M., Palmer D.H., Edeline J., Abou-Alfa G.K., Cheng A.-L., Decaens T., El-Khoueiry A.B., Finn R.S. (2021). A Phase III, Double-Blind, Randomized Study of Nivolumab (NIVO) and Ipilimumab (IPI), Nivo Monotherapy or Placebo plus Transarterial Chemoembolization (TACE) in Patients with Intermediate-Stage Hepatocellular Carcinoma (HCC). J. Clin. Oncol..

[78] Llovet J.M., Vogel A., Madoff D.C., Finn R.S., Ogasawara S., Ren Z., Mody K., Li J.J., Siegel A.B., Dubrovsky L. (2022). Randomized Phase 3 LEAP-012 Study: Transarterial Chemoembolization with or Without Lenvatinib Plus Pembrolizumab for Intermediate-Stage Hepatocellular Carcinoma Not Amenable to Curative Treatment. Cardiovasc. Intervent Radiol..

[79] Kloeckner R., Galle P.R., Bruix J. (2021). Local and Regional Therapies for Hepatocellular Carcinoma. Hepatology.

[80] Kudo M., Guo Y., Hua Y., Zhao M., Xing W., Zhang Y., Liu R., Ren Z., Gu S., Lin Z. (2022). TALENTACE: A Phase III, Open-Label, Randomized Study of on-Demand Transarterial Chemoembolization Combined with Atezolizumab + Bevacizumab or on-Demand Transarterial Chemoembolization Alone in Patients with Untreated Hepatocellular Carcinoma. J. Clin. Oncol..

[81] Foerster F., Kloeckner R., Reig M., Chan S.L., Chung J.W., Merle P., Park J.-W., Piscaglia F., Vogel A., Gaillard V. (2022). ABC-HCC: A Phase IIIb, Randomized, Multicenter, Open-Label Trial of Atezolizumab plus Bevacizumab versus Transarterial Chemoembolization (TACE) in Intermediate-Stage Hepatocellular Carcinoma. J. Clin. Oncol..

[82] Kudo M. (2022). Atezolizumab plus Bevacizumab Followed by Curative Conversion (ABC Conversion) in Patients with Unresectable, TACE-Unsuitable Intermediate-Stage Hepatocellular Carcinoma. Liver Cancer.

[83] Reck M., Rodríguez-Abreu D., Robinson A.G., Hui R., Csőszi T., Fülöp A., Gottfried M., Peled N., Tafreshi A., Cuffe S. (2016). Pembrolizumab versus Chemotherapy for PD-L1–Positive Non–Small-Cell Lung Cancer. N. Engl. J. Med..

[84] Davis A.A., Patel V.G. (2019). The Role of PD-L1 Expression as a Predictive Biomarker: An Analysis of All US Food and Drug Administration (FDA) Approvals of Immune Checkpoint Inhibitors. J. Immunother. Cancer.

[85] Iwata H., Emens L., Adams S., Barrios C.H., Diéras V., Loi S., Rugo H.S., Schneeweiss A., Winer E.P., Patel S. (2020). 49MO IMpassion130: Final OS Analysis from the Pivotal Phase III Study of Atezolizumab + Nab-Paclitaxel vs Placebo + Nab-Paclitaxel in Previously Untreated Locally Advanced or Metastatic Triple-Negative Breast Cancer. Ann. Oncol..

[86] Zhang X., Ning C., Zhao H. (2023). Tissue-Based PD-L1 Expression Is the Strongest Predictor of Overall Survival Benefit from ICI in Advanced Gastroesophageal Cancer. JAMA Oncol..

[87] Doroshow D.B., Bhalla S., Beasley M.B., Sholl L.M., Kerr K.M., Gnjatic S., Wistuba I.I., Rimm D.L., Tsao M.S., Hirsch F.R. (2021). PD-L1 as a Biomarker of Response to Immune-Checkpoint Inhibitors. Nat. Rev. Clin. Oncol..

[88] Pinato D.J., Mauri F.A., Spina P., Cain O., Siddique A., Goldin R., Victor S., Pizio C., Akarca A.U., Boldorini R.L. (2019). Clinical Implications of Heterogeneity in PD-L1 Immunohistochemical Detection in Hepatocellular Carcinoma: The Blueprint-HCC Study. Br. J. Cancer.

[89] Melero I., Neely J., Sangro B., Finn R.S., Abou-Alfa G.K., Cheng A.-L., Yau T., Furuse J., Park J.-W., Wadhawan S. (2019). Biomarkers and Clinical Outcomes in Nivolumab-Treated Patients with Advanced Hepatocellular Carcinoma in CheckMate 040. Ann. Oncol..

[90] Sangro B., Melero I., Wadhawan S., Finn R.S., Abou-Alfa G.K., Cheng A.-L., Yau T., Furuse J., Park J.-W., Boyd Z. (2020). Association of Inflammatory Biomarkers with Clinical Outcomes in Nivolumab-Treated Patients with Advanced Hepatocellular Carcinoma. J. Hepatol..

[91] Zhu A.X., Finn R.S., Edeline J., Cattan S., Ogasawara S., Palmer D., Verslype C., Zagonel V., Fartoux L., Vogel A. (2018). Pembrolizumab in Patients with Advanced Hepatocellular Carcinoma Previously Treated with Sorafenib (KEYNOTE-224): A Non-Randomised, Open-Label Phase 2 Trial. Lancet Oncol..

[92] Yang Y., Chen D., Zhao B., Ren L., Huang R., Feng B., Chen H. (2023). The Predictive Value of PD-L1Expression in Patients with Advanced Hepatocellular Carcinoma Treated with PD -1/PD-L1 Inhibitors: A Systematic Review and Meta-analysis. Cancer Med..

[93] Sha D., Jin Z., Budczies J., Kluck K., Stenzinger A., Sinicrope F.A. (2020). Tumor Mutational Burden as a Predictive Biomarker in Solid Tumors. Cancer Discov..

[94] Wong M., Kim J.T., Cox B., Larson B.K., Kim S., Waters K.M., Vail E., Guindi M. (2021). Evaluation of Tumor Mutational Burden in Small Early Hepatocellular Carcinoma and Progressed Hepatocellular Carcinoma. Hepat. Oncol..

[95] Muhammed A., D’Alessio A., Enica A., Talbot T., Fulgenzi C.A.M., Nteliopoulos G., Goldin R.D., Cortellini A., Pinato D.J. (2022). Predictive Biomarkers of Response to Immune Checkpoint Inhibitors in Hepatocellular Carcinoma. Expert. Rev. Mol. Diagn..

[96] Eso Y., Shimizu T., Takeda H., Takai A., Marusawa H. (2020). Microsatellite Instability and Immune Checkpoint Inhibitors: Toward Precision Medicine against Gastrointestinal and Hepatobiliary Cancers. J. Gastroenterol..

[97] Kawaoka T., Ando Y., Yamauchi M., Suehiro Y., Yamaoka K., Kosaka Y., Fuji Y., Uchikawa S., Morio K., Fujino H. (2020). Incidence of Microsatellite Instability-high Hepatocellular Carcinoma among Japanese Patients and Response to Pembrolizumab. Hepatol. Res..

[98] Heimbach J.K., Kulik L.M., Finn R.S., Sirlin C.B., Abecassis M.M., Roberts L.R., Zhu A.X., Murad M.H., Marrero J.A. (2018). AASLD Guidelines for the Treatment of Hepatocellular Carcinoma. Hepatology.

[99] Brummel K., Eerkens A.L., de Bruyn M., Nijman H.W. (2023). Tumour-Infiltrating Lymphocytes: From Prognosis to Treatment Selection. Br. J. Cancer.

[100] Thomas N.E., Busam K.J., From L., Kricker A., Armstrong B.K., Anton-Culver H., Gruber S.B., Gallagher R.P., Zanetti R., Rosso S. (2013). Tumor-Infiltrating Lymphocyte Grade in Primary Melanomas Is Independently Associated with Melanoma-Specific Survival in the Population-Based Genes, Environment and Melanoma Study. J. Clin. Oncol..

[101] Zeng D.-Q., Yu Y.-F., Ou Q.-Y., Li X.-Y., Zhong R.-Z., Xie C.-M., Hu Q.-G. (2016). Prognostic and Predictive Value of Tumor-Infiltrating Lymphocytes for Clinical Therapeutic Research in Patients with Non-Small Cell Lung Cancer. Oncotarget.

[102] Presti D., Dall’Olio F.G., Besse B., Ribeiro J.M., Di Meglio A., Soldato D. (2022). Tumor Infiltrating Lymphocytes (TILs) as a Predictive Biomarker of Response to Checkpoint Blockers in Solid Tumors: A Systematic Review. Crit. Rev. Oncol. Hematol..

[103] Macek Jilkova Z., Aspord C., Kurma K., Granon A., Sengel C., Sturm N., Marche P.N., Decaens T. (2019). Immunologic Features of Patients with Advanced Hepatocellular Carcinoma Before and During Sorafenib or Anti-Programmed Death-1/Programmed Death-L1 Treatment. Clin. Transl. Gastroenterol..

[104] Ng H.H.M., Lee R.Y., Goh S., Tay I.S.Y., Lim X., Lee B., Chew V., Li H., Tan B., Lim S. (2020). Immunohistochemical Scoring of CD38 in the Tumor Microenvironment Predicts Responsiveness to Anti-PD-1/PD-L1 Immunotherapy in Hepatocellular Carcinoma. J. Immunother. Cancer.

[105] Hong J.Y., Cho H.J., Sa J.K., Liu X., Ha S.Y., Lee T., Kim H., Kang W., Sinn D.H., Gwak G.-Y. (2022). Hepatocellular Carcinoma Patients with High Circulating Cytotoxic T Cells and Intra-Tumoral Immune Signature Benefit from Pembrolizumab: Results from a Single-Arm Phase 2 Trial. Genome Med..

[106] Haber P.K., Castet F., Torres-Martin M., Andreu-Oller C., Puigvehí M., Miho M., Radu P., Dufour J.-F., Verslype C., Zimpel C. (2023). Molecular Markers of Response to Anti-PD1 Therapy in Advanced Hepatocellular Carcinoma. Gastroenterology.

[107] Zhu A.X., Abbas A.R., de Galarreta M.R., Guan Y., Lu S., Koeppen H., Zhang W., Hsu C.-H., He A.R., Ryoo B.-Y. (2022). Molecular Correlates of Clinical Response and Resistance to Atezolizumab in Combination with Bevacizumab in Advanced Hepatocellular Carcinoma. Nat. Med..

[108] Harding J.J., Nandakumar S., Armenia J., Khalil D.N., Albano M., Ly M., Shia J., Hechtman J.F., Kundra R., El Dika I. (2019). Prospective Genotyping of Hepatocellular Carcinoma: Clinical Implications of Next-Generation Sequencing for Matching Patients to Targeted and Immune Therapies. Clin. Cancer Res..

[109] Haber P.K., Puigvehí M., Castet F., Lourdusamy V., Montal R., Tabrizian P., Buckstein M., Kim E., Villanueva A., Schwartz M. (2021). Evidence-Based Management of Hepatocellular Carcinoma: Systematic Review and Meta-Analysis of Randomized Controlled Trials (2002–2020). Gastroenterology.

[110] Lee C.-K., Chan S.L., Chon H.J. (2022). Could We Predict the Response of Immune Checkpoint Inhibitor Treatment in Hepatocellular Carcinoma?. Cancers.

[111] Pfister D., Núñez N.G., Pinyol R., Govaere O., Pinter M., Szydlowska M., Gupta R., Qiu M., Deczkowska A., Weiner A. (2021). NASH Limits Anti-Tumour Surveillance in Immunotherapy-Treated HCC. Nature.

[112] Kudo M. (2021). Lack of Response to Immunotherapy in Non-Alcoholic Steatohepatitis Related Hepatocellular Carcinoma. Hepatobiliary Surg. Nutr..

[113] Qin S., Chan L.S., Gu S., Bai Y., Ren Z., Lin X., Chen Z., Jia W., Jin Y., Guo Y. (2022). LBA35 Camrelizumab (C) plus Rivoceranib (R) vs. Sorafenib (S) as First-Line Therapy for Unresectable Hepatocellular Carcinoma (UHCC): A Randomized, Phase III Trial. Ann. Oncol..

[114] Chan S.L., Kudo M., Sangro B., Kelley R.K., Furuse J., Park J.-W., Sunpaweravong P., Fasolo A., Yau T., Kawaoka T. (2022). 83P Impact of Viral Aetiology in the Phase III HIMALAYA Study of Tremelimumab (T) plus Durvalumab (D) in Unresectable Hepatocellular Carcinoma (UHCC). Ann. Oncol..

[115] D’Alessio A., Fulgenzi C.A.M. (2021). Treating Patients with Advanced Hepatocellular Carcinoma and Impaired Liver Function: Broadening the Reach of Anti-cancer Therapy. Liver Cancer Int..

[116] Fessas P., Kaseb A., Wang Y., Saeed A., Szafron D., Jun T., Dharmapuri S., Rafeh Naqash A., Muzaffar M., Navaid M. (2020). Post-Registration Experience of Nivolumab in Advanced Hepatocellular Carcinoma: An International Study. J. Immunother. Cancer.

[117] Haanen J., Obeid M., Spain L., Carbonnel F., Wang Y., Robert C., Lyon A.R., Wick W., Kostine M., Peters S. (2022). Management of Toxicities from Immunotherapy: ESMO Clinical Practice Guideline for Diagnosis, Treatment and Follow-Up. Ann. Oncol..

[118] Pinter M., Scheiner B., Peck-Radosavljevic M. (2021). Immunotherapy for Advanced Hepatocellular Carcinoma: A Focus on Special Subgroups. Gut.

[119] Kennedy L.C., Bhatia S., Thompson J.A., Grivas P. (2019). Preexisting Autoimmune Disease: Implications for Immune Checkpoint Inhibitor Therapy in Solid Tumors. J. Natl. Compr. Cancer Netw..

[120] Shi X.-L., Mancham S., Hansen B.E., de Knegt R.J., de Jonge J., van der Laan L.J.W., Rivadeneira F., Metselaar H.J., Kwekkeboom J. (2016). Counter-Regulation of Rejection Activity against Human Liver Grafts by Donor PD-L1 and Recipient PD-1 Interaction. J. Hepatol..

